# Aortic valve stenosis and physical activity at high altitude in a female trekker: sex-specific considerations and the added value of a sports cardiology team—a case report

**DOI:** 10.1093/ehjcr/ytag593

**Published:** 2026-08-17

**Authors:** Mathijs Bodde, Danny van de Sande, Philippine Kiès, Nick Bijsterveld, Harald Jorstad

**Affiliations:** Department of Cardiology, Amsterdam Cardiovascular Sciences, Amsterdam Movement Sciences, Amsterdam University Medical Center, Postbox 22660, 1100 DD Amsterdam, The Netherlands; Department of Cardiology, Maxima Medical Center, Postbox 7777, 5500 MB Veldhoven, The Netherlands; Department of Cardiology, Leiden University Medical Center, Postbox 9600, 2300 RC Leiden, The Netherlands; Department of Cardiology, Amsterdam Cardiovascular Sciences, Amsterdam Movement Sciences, Amsterdam University Medical Center, Postbox 22660, 1100 DD Amsterdam, The Netherlands; Department of Cardiology, Amsterdam Cardiovascular Sciences, Amsterdam Movement Sciences, Amsterdam University Medical Center, Postbox 22660, 1100 DD Amsterdam, The Netherlands

**Keywords:** Aortic valve disease, High-altitude, Sports cardiology, Multidisciplinary team, Biological sex, Case report

## Abstract

**Background:**

Recommendations for high-altitude exposure in individuals with cardiovascular disease are largely based on expert opinion. This report details sport- and sex-specific recommendations for a patient with moderate aortic valve stenosis preparing for a high-altitude trek.

**Case summary:**

A 64-year-old woman with moderate, asymptomatic aortic valve stenosis and excellent functional capacity sought sports advice before a planned trek in the Himalayas. After a multidisciplinary sports cardiology team discussion and considering limited data on sex-specific cardiovascular responses to hypoxia, the trek was deemed safe with specific precautions. The patient was advised to limit activity to moderate intensity (74% of maximal heart rate), start at low altitude, and gradually ascend, not exceeding 2500 m and to descend immediately should symptoms occur. She was also counselled on the potential influence of sex hormones on exercise tolerance at high altitude. She successfully completed the trek without incident. Follow-up after 6 months showed progression to severe aortic stenosis, for which she underwent aortic valve and root replacement.

**Discussion:**

This case demonstrates that, following comprehensive evaluation, a high-altitude trek may be feasible in selected patients with moderate aortic stenosis. It highlights the need for individualized advice, taking into account physiological responses to high-altitude exposure, and emphasizes potential sex-specific considerations, despite limited data.

Learning points

- High-altitude trek appears to be safe and feasible after a comprehensive pre-exposure assessment and recommendations for patients with moderate aortic valve stenosis
- A multidisciplinary sports cardiology team can optimize the formulation of tailored sports advice with a low cardiovascular event rate, complementing existing guidelines
- Sex-related differences in the physiological response to hypoxia in patients with aortic valve stenosis exist; however, further research is warranted to unravel these differences

## Introduction

More than 5 million people aged over 60 years visit high altitudes (>2000–2500 m) each year.^1^ At high altitude, hypoxaemia triggers sympathetic activation to maintain oxygenation, resulting in increased heart rate, cardiac output, coronary blood flow, and systemic and pulmonary pressures.^2^ This response may be particularly challenging for individuals with cardiovascular disease (CVD) and limited functional reserve, especially women, who may experience greater declines in arterial oxygen saturation (SpO_2_) due to mechanical ventilatory limitations, potentially resulting in a lower VO_2_peak.^3^

Aortic stenosis (AS) is the most common valvular heart disease in developed countries and is often age related.^4^ Disease progression increases the transvalvular pressure gradient and left ventricular (LV) workload, leading to LV hypertrophy and increased myocardial oxygen demand. Severe AS increases the risk of heart failure and sudden cardiac death due to mechanical obstruction, arrhythmias, or coronary hypoperfusion.^5^

Current recommendations regarding high-altitude exposure in patients with CVD are largely based on expert opinion.^6^ Multidisciplinary sports cardiology teams (MDTs) provide complementary expertise and enable tailored exercise recommendations, thereby reducing unnecessary restrictions while maintaining a low cardiovascular event rate, in line with existing guidelines.^7^ This case report describes how sport-specific recommendations enabled a woman with moderate AS to participate safely in a high-altitude trek.

## Summary figure

**Figure:**
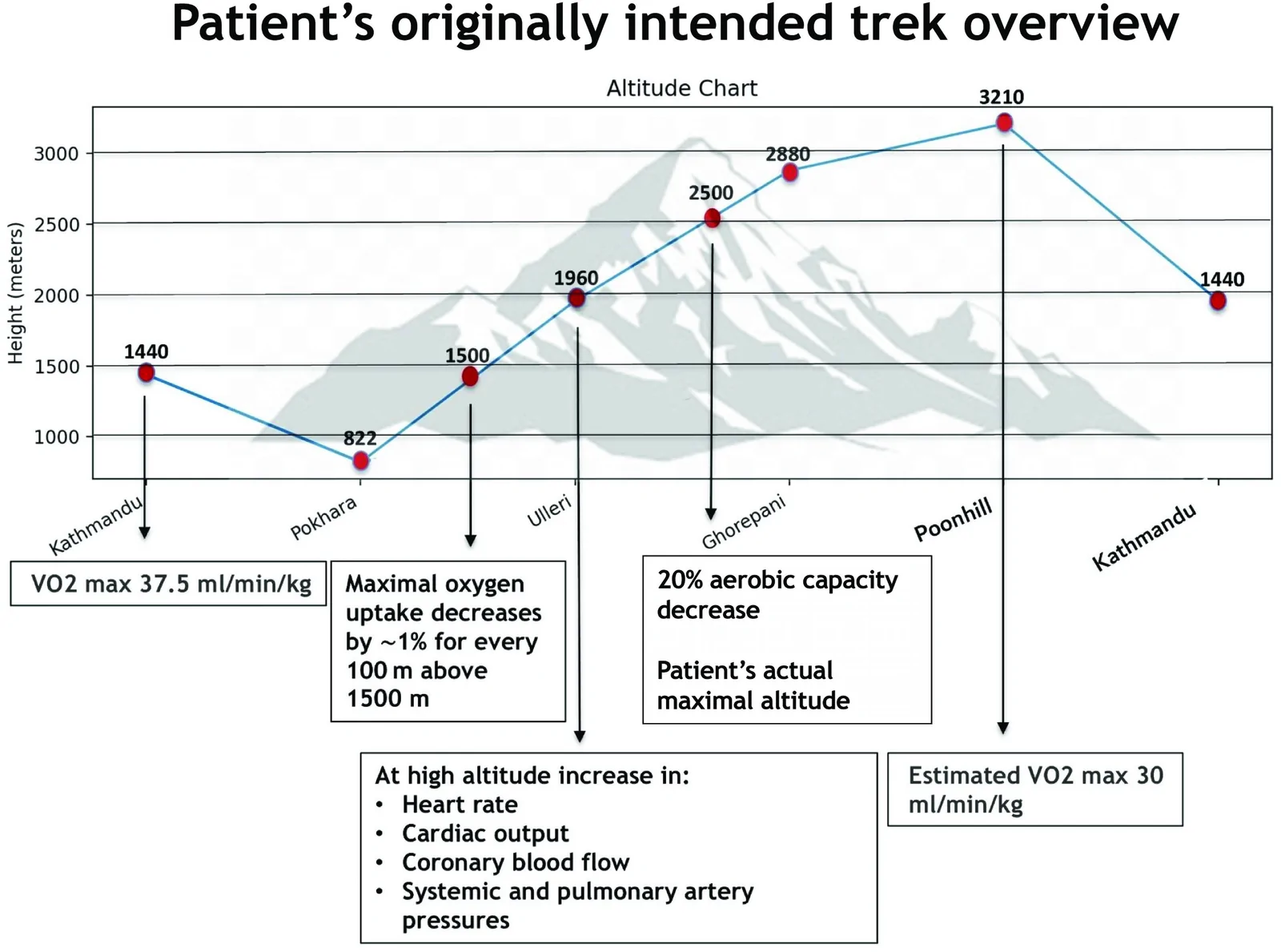


## Case presentation

A 64-year-old Caucasian woman, a former smoker (stopped 30 years ago), with a BMI of 24.7 kg/m^2^, was referred to the cardiology outpatient clinic for evaluation after an episode of tachycardia during training for a trek in the Himalayas. Her medical history included exercise-induced asthma, obstructive sleep apnoea syndrome (OSAS) treated with continuous positive airway pressure, and depression. Her medication consisted of clomipramine and budesonide/formoterol inhalers. She reported no use of dietary supplements or ergogenic aids. She exercised regularly at a gym using a cross-trainer and step machine two to three times per week for 1 h at moderate intensity.

**Figure 1:**
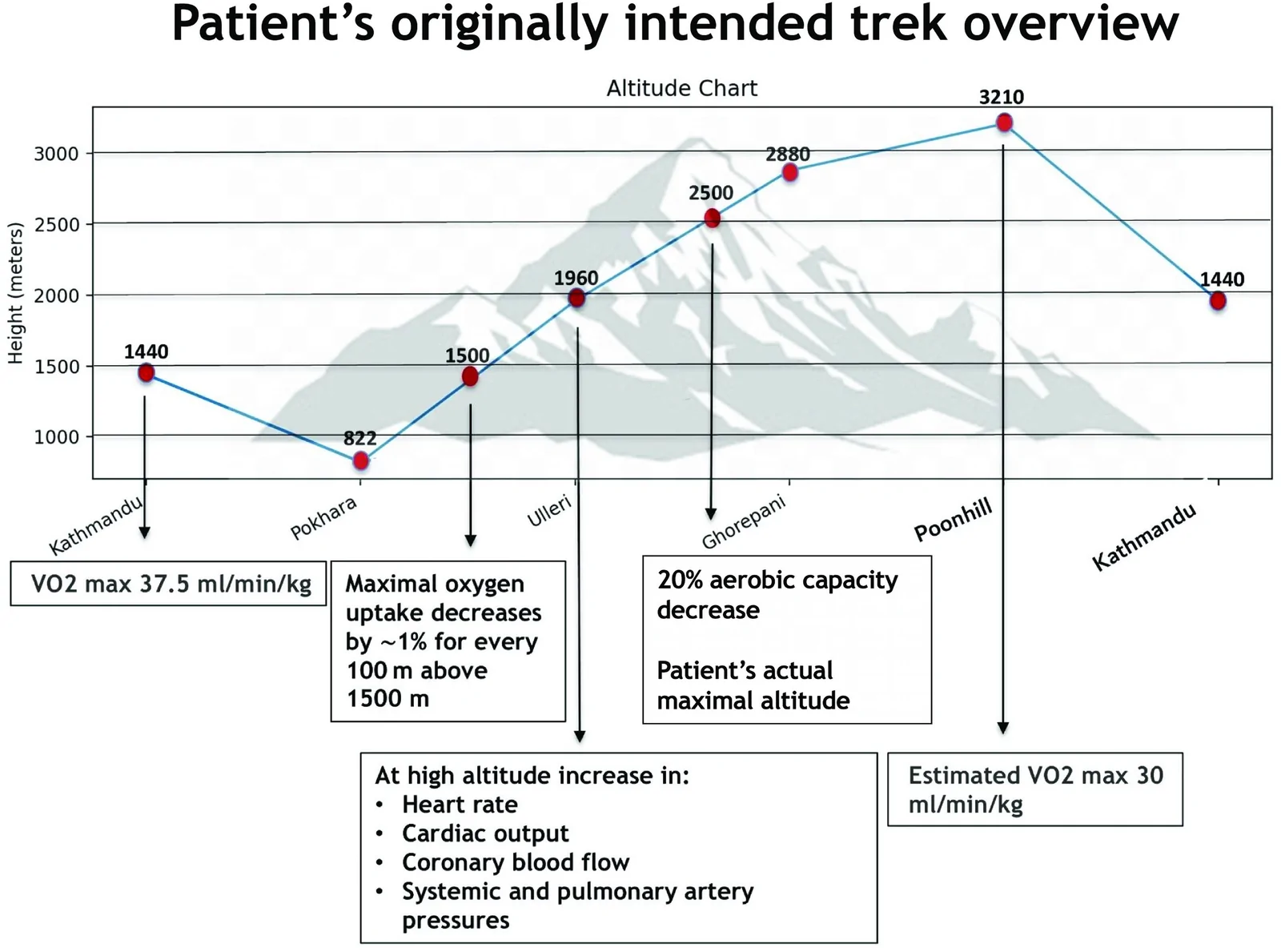
Patient’s originally intented schematic overview of the 5-day Poon Hill Trek with physiological changes at high altitude. Patients actual route did not exceed 2500 m.

The originally intended planned trek in the Himalayas involved a maximum altitude of 3300 m, a base camp at 1500 m, daily trekking for 5–6 h, and porter support for carrying equipment (*Figure 1*).

**Figure 2:**
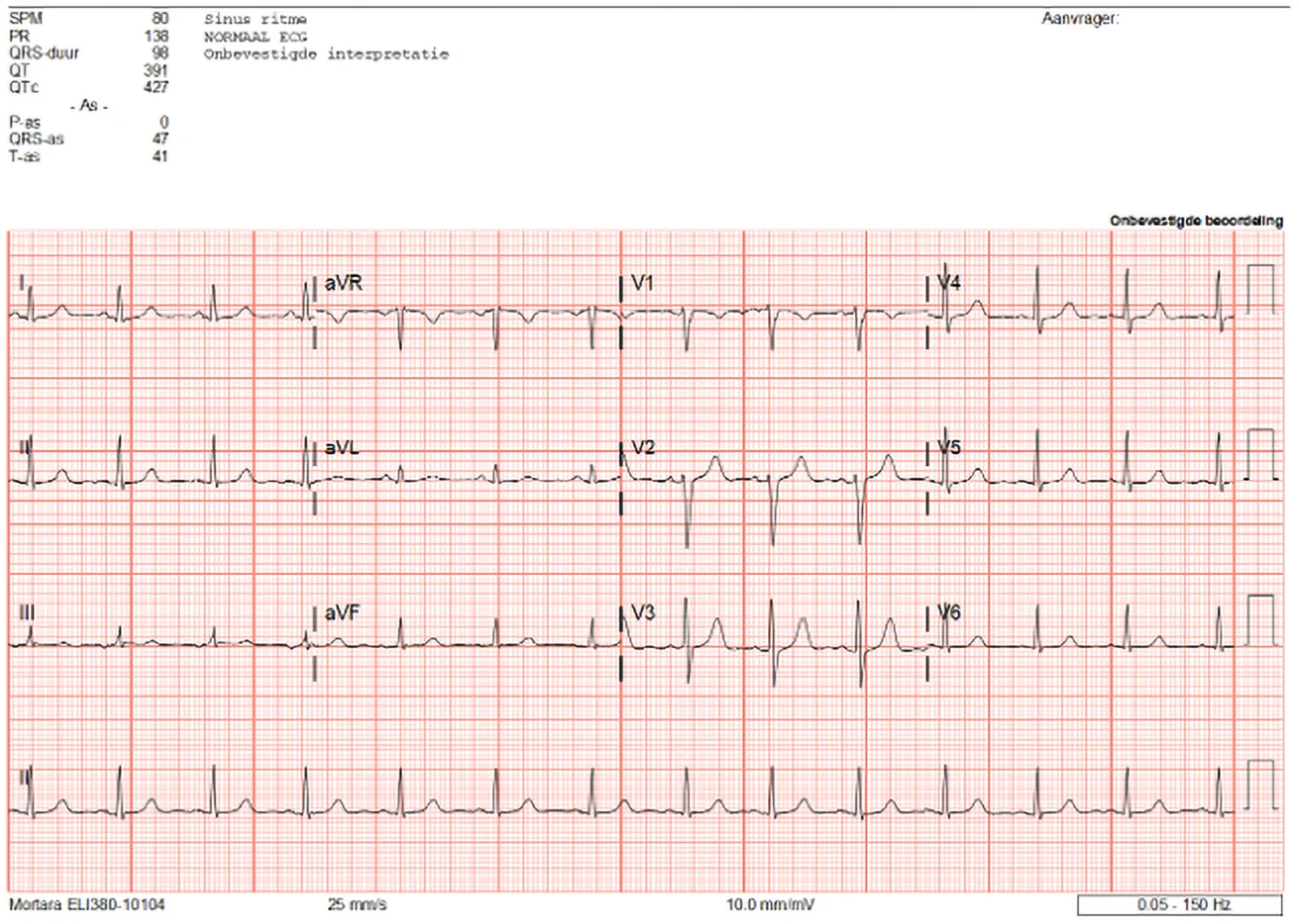
Outpatient clinic electrocardiogram-sinus rhythm with normal axis, normal conduction times and no ST-T segment changes, and normal voltages.

At the outpatient clinic, her blood pressure was 105/60 mmHg, and her resting heart rate was 80 beats/min. Clinical examination revealed a systolic murmur (Grade 2/6), best heard over the left ventricular outflow tract and radiating to the carotid arteries. Electrocardiography showed normal sinus rhythm at 80 beats/min (*Figure 2*). A 24-h ambulatory electrocardiogram showed no abnormalities.

Exercise testing demonstrated a maximal workload of 185 W (170% of predicted), an estimated VO_2_max of 37.5 mL/kg/min, and a maximal heart rate of 160 beats/min (98% of predicted). The blood pressure response was blunted, increasing from 121/88 mmHg at rest to 148/98 mmHg during maximal exercise. The patient reported no symptoms, and no ST-segment changes were observed during exercise.

**Figure 3:**
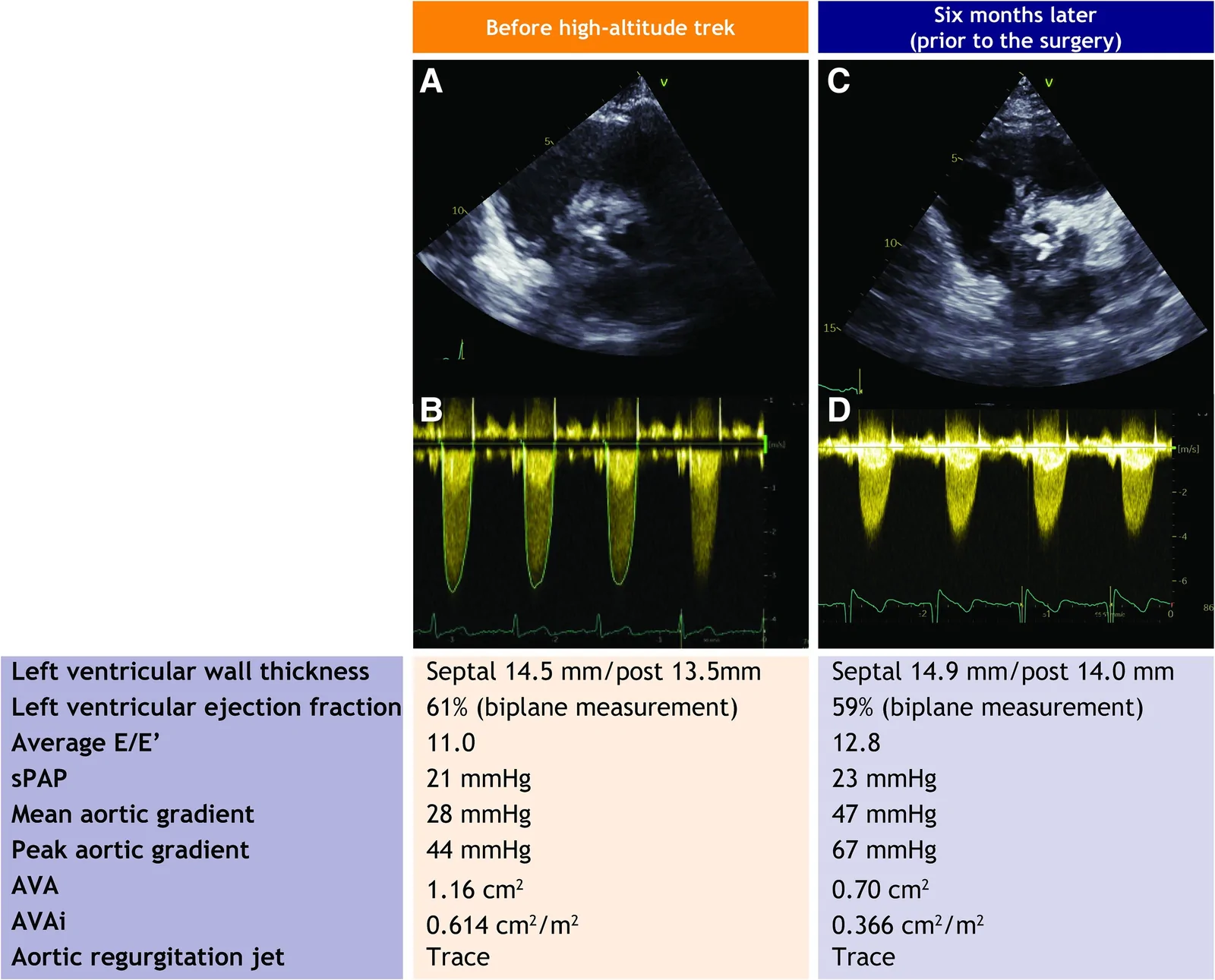
Transthoracic echocardiogram before high-altitude trek (*A* and *B*) and after 6 months of follow-up (*C* and *D*). Corresponding echocardiographic measurements are shown below each time point. (*A*) Two-dimensional parasternal short axis of the aortic valve with a bicuspid valve with moderate calcification. (*B*) Continuous-wave Doppler across the aortic valve with a mean and peak aortic gradient (28 and 44 mmHg respectively). (*C*) Two-dimensional parasternal short axis of the aortic valve with a bicuspid valve with severe calcification. (*D*) Continuous-wave Doppler across the aortic valve with a mean and peak aortic gradient (47 and 67 mmHg respectively). sPAP, systolic pulmonary artery pressure; AVA, aortic valve area; AVAi, aortic valve area indexed; post, posterior.

Echocardiography showed concentric left ventricular hypertrophy with a normal ejection fraction. A bicuspid aortic valve with moderate stenosis was present, with a calculated aortic valve area of 1.16 cm^2^, a peak gradient of 44 mmHg, and a mean gradient of 28 mmHg with a trace aortic regurgitation (*Figure 3*).

Computed tomography demonstrated an aortic root diameter of 39 mm and a mid-ascending aortic diameter of 44 mm.

The case was discussed by the national Sports Cardiology multidisciplinary panel.^7^ As the patient was asymptomatic with excellent exercise capacity, the planned trek was considered safe, provided that specific precautions were taken, while acknowledging the limited data on sex differences in responses to hypoxia. In line with the ESC sports cardiology guidelines, the patient was advised not to exceed moderate-intensity physical activity, with a target heart rate of 120 beats/min, corresponding to 74% of maximal heart rate (recommended range, 55%–74%).^7^

The patient was advised to begin at low altitude (<1500 m) and to ascend gradually above 2000 m, and she was also advised not to exceed 2500 m and to descend immediately if symptoms occurred.^8^

In addition, the patient was informed about the limited data regarding sex differences in responses to hypoxia and the potential for a greater decline in arterial SpO_2_ and VO_2_peak in women compared with men.^3^

Obstructive sleep apnoea syndrome and exercise-induced asthma are associated with an increased risk of high-altitude illness and pulmonary hypertension. Since being diagnosed with OSAS, the patient had lost 9 kg. A repeat sleep study showed an apnoea–hypopnoea index of two events per hour while using a mandibular repositioning device. Therefore, significant pulmonary complications were not expected.

The patient adhered to these recommendations, did not exceed 2500 m, and completed the trek without adverse events. However, 6 months later, she reported exertional fatigue during a low-altitude hike. Follow-up echocardiography showed progression to severe AS (aortic valve area, 0.7 cm^2^; mean gradient, 47 mmHg) (*Figure 3*). The patient subsequently underwent successful aortic valve and root replacement, and her recovery was uneventful.

## Discussion

In asymptomatic individuals with moderate AS, the European Society of Cardiology sports cardiology guidelines permit participation in low-to-moderate intensity recreational sports, provided that left ventricular ejection fraction is >50%, functional capacity is preserved, and exercise testing is normal. Specific recommendations regarding high-altitude exposure in this population are lacking, and evidence on valvular heart disease at altitude remains limited.^6^

Patients with cardiovascular disease should seek counselling before travelling to high altitude. Prerequisites include clinical stability, absence of symptoms at rest, and adequate functional capacity. In moderate AS, echocardiography and standard exercise testing—assessing functional capacity, ischaemia, arrhythmias, and blood pressure response (>20 systolic mmHg increase)—are generally sufficient for risk stratification. Although in this case the systolic blood pressure response was not formally abnormal,^6^ it was somewhat blunted, likely reflecting the high transvalvular gradient limiting cardiac output augmentation.^9^ In severe AS, high-altitude exposure is contraindicated. However, asymptomatic patients with preserved ejection fraction may undertake air travel, as cabin pressure corresponds to an altitude of ∼1800–2500 m (see *Table 1*).

**Table 1:** Risk factors, prerequisites, and recommendations for altitude travel in patients with aortic stenosis

Category	Details
Risk factors for sudden cardiac death (SCD)	First day at altitude Late morning During or immediately after unaccustomed physical exertion Presence of cardiovascular risk factors Physiological stress (e.g. anxiety, poor sleep, intercurrent infection, inadequate caloric intake, glycogen depletion, and dehydration)
General prerequisites (low altitude)	Stable clinical condition Asymptomatic at rest Adequate physical conditioning prior to travel
Pre-travel evaluation	Transthoracic echocardiography (TTE) Symptom-limited exercise testing Consider cardiopulmonary exercise testing (CPET)
General recommendations	Gradual ascent above 2000 m (300–500 m/day) Avoid rapid ascent to >3000 m Avoid excessive physical exertion Maintain hydration; treat losses promptly Ensure blood pressure control New-onset arrhythmia → immediate descent Descend if symptoms occur Continue prescribed medication

Practical recommendations according to severity of aortic stenosis	
Severity	Recommendations
Mild aortic stenosis	No restrictions regarding exercise or altitude exposure
Moderate aortic stenosis	If LVEF ≥ 50% and good functional capacity: Moderate-intensity exercise only (based on BORG score, %HRmax, or talk test) Maximum altitude ∼2500 m 2500–3000 m may be considered (limited evidence)
Severe or symptomatic aortic stenosis	High-altitude exposure contraindicated Travel (by car or plane) < 2500 m may be considered if asymptomatic and stable

Adapted from: European Society of Cardiology guidelines and expert consensus on cardiovascular disease and high-altitude exposure.

SCD, sudden cardiac death; LVEF, left ventricular ejection fraction; TTE, transthoracic echocardiography; CPET, cardiopulmonary exercise testing.

A multidisciplinary team (MDT) can further individualize exercise recommendations. It includes expertise in sports cardiology, cardiovascular imaging, and sports and exercise medicine, with additional specialists consulted as needed.

In this case, the pragmatic recommendation not to exceed 2500 m was based on physiological considerations and the available, limited literature. At this altitude, inspired oxygen pressure decreases by ∼25%, triggering increases in heart rate, contractility, and cardiac output to maintain oxygen delivery, thereby increasing myocardial workload and oxygen demand.^10, 11^ Small observational studies in patients with cardiovascular disease suggest additional physiological stress at altitude.^8, 12^

Moreover, sex-specific differences in high-altitude responses have been described. Compared with men, women may experience greater reductions in SpO_2_ and VO_2_peak, possibly due to ventilatory limitations.^3, 13^ These responses are influenced by hormonal fluctuations; e.g. oestrogen may enhance hypoxic pulmonary vasoconstriction and reduce heart rate, potentially impairing haemodynamics and further reducing VO_2_peak.^3, 13^

Burtscher^14^ reported that prolonged hiking requires an oxygen consumption of ∼18 mL/kg/min, corresponding to about 62% of VO_2_max. Consequently, a VO_2_max of around 30 mL/kg/min is required to sustain a normal ascent rate (*Figure 1*).

Since VO_2_max declines by ∼1% per 100 m above 1500 m,^15^ a VO_2_max of ∼33 mL/kg/min would be required at 2500 m. In the present case, the estimated VO_2_max was 37.5 mL/kg/min, suggesting sufficient aerobic capacity for the planned altitude.

Additional factors, such as heavy footwear and adverse weather conditions, may further increase physiological demands.^16^

A limitation of this case report is the use of standard exercise testing rather than cardiopulmonary exercise testing, which would allow more precise prescription based on ventilatory thresholds (VT1 and VT2). Cardiopulmonary exercise testing could also provide additional insight into the functional severity of AS through oxygen pulse analysis.^17, 18^

In conclusion, following comprehensive assessment and MDT discussion, a high-altitude trek may be feasible in selected patients with moderate AS. Recommendations should consider both disease severity and altitude-related physiological responses, including potential sex-specific effects, while recognizing current evidence limitations.

## Data Availability

The data underlying this article will be shared on reasonable request to the corresponding author.

## Lead author biography

Mathijs Bodde, MD, PhD, is a fellow in Sports Cardiology at the Department of Cardiology, Amsterdam University Medical Center, Amsterdam, the Netherlands. He obtained his PhD in 2020 and graduated from the Faculty of Medicine at Leiden University Medical Center in 2025. His clinical and research interests include sports cardiology, preventive cardiology, and cardiovascular imaging.

## Notes

**Consent:** Patient consent for case report publication is obtained in compliance with the COPE guidelines.
